# Interplay Between Autophagy, Cellular Senescence, and Brain Aging: Neuroprotective Implications of Intermittent Fasting

**DOI:** 10.1007/s10571-026-01709-7

**Published:** 2026-03-11

**Authors:** Ishika Singh, Shreya Bhat, Rajesh Tamatta, Abhishek Kumar Singh

**Affiliations:** Department of Biotherapeutics Research, Manipal Academy of Higher Education, Manipal, Karnataka 576104 India

**Keywords:** Aging, Senescence, Autophagy, Dietary restriction, Intermittent fasting

## Abstract

Aging is characterized by progressive deterioration in cellular function and molecular integrity, which in turn increases vulnerability to age-related diseases, such as neurodegenerative disorders. Cellular senescence is a hallmark of biological aging that plays a crucial role in the development of neurodegenerative diseases. Cellular autophagy, a lysosome-mediated process of degradation, is crucial for maintaining cellular homeostasis and longevity. Nutritional strategies, such as intermittent fasting (IF), which consist of cycles of fasting and normal feeding, have been recognized as potential methods to induce autophagy and derive health benefits. Autophagy is crucial for the degradation of sequestered proteins and damaged organelles, thus maintaining cellular vigor and balance. However, with increasing age, autophagy is compromised, suggesting the need for a stable or optimal rate of autophagy for the maintenance of cellular uptake. IF has the potential to modulate the process of autophagy by inducing changes in ATP and ADP levels during fasting through the activation of pathways such as the AMPK and Sirtuin 1 pathways, which promote the activation of autophagosome formation while simultaneously inhibiting mTOR, an inhibitor of autophagy. Despite the encouraging properties of the IF, there are constraints, as responses may vary incredibly among subjects, and the ideal duration of fasting is uncertain. Furthermore, the potential for new metabolic diseases associated with IF is not fully understood. Understanding the intricate relationships among aging, autophagy, and dietary restrictions such as intermittent fasting could pave the way for novel therapeutic strategies to increase longevity and mitigate age-related health issues.

**Graphical Abstract:**

Brain aging is associated with various hallmarks leading to neurodegenerative diseases and impaired autophagy, causing the accumulation of senescent cells. Intermittent fasting, a dietary regimen, controls food intake by alternating between eating and fasting. It regulates various pathways that facilitate autophagy activation and senescent cells clearance in the brain

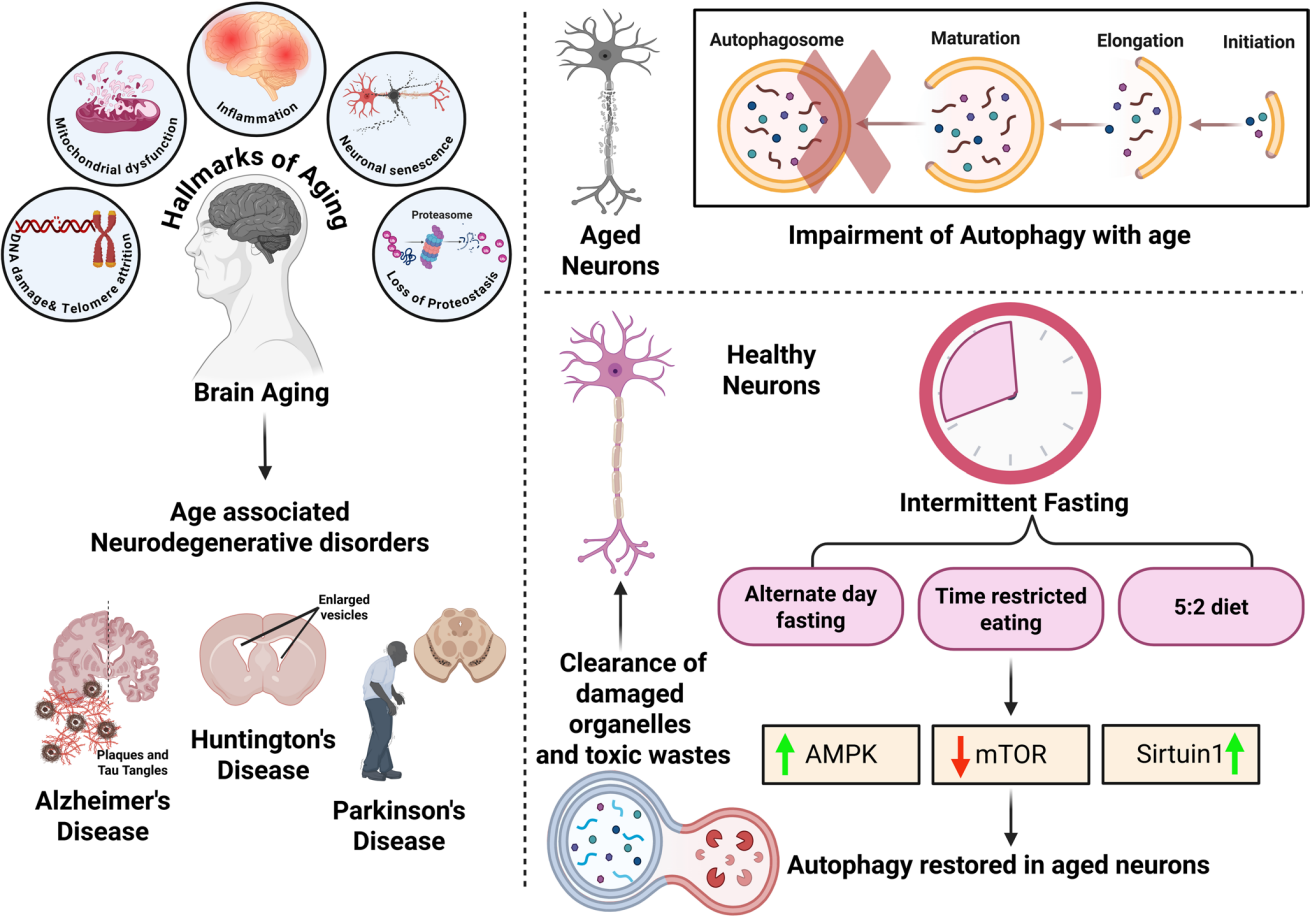

## Introduction

Aging is a ubiquitous process that affects all living things and poses a difficult problem for organisms. As we grow older, our bodies work less well, making us more likely to get sick and die. Scientists see aging in many ways (Guo et al. 2022). When we age, our bodies do not function as efficiently. For example, our hearts might not beat, our cognitive abilities may decline, and our immune system might have difficulty fighting infections. From an evolutionary perspective, aging makes an organism less fit. This fitness refers to how well we can live and produce offspring, which worsens as our bodies weaken and our body processes slow down (da Costa et al. 2016). Our genes and where we live play a large part in how long we live, but eating well and working out can help slow down the effects of getting older that we cannot avoid.

The primary hallmarks of aging include DNA damage built up over time, caused by genomic instability. Telomere attrition occurs when the telomere becomes shorter, causing cells to stop dividing. There is a change in gene expression, and proteins do not fold or break down, leading to a loss of proteostasis. Mitochondrial dysfunction occurs, affecting how cells generate energy. Cell-to-cell signaling pathways break down, altering intercellular communication. Moreover, long-lasting inflammation occurs in these tissues. Knowing about these signs helps in understanding how aging works and points to possible ways to treat ARDs (Barbosa et al. 2019; López-Otín et al. 2023). Cellular senescence is a hallmark of biological aging that plays a crucial role in the development of neurodegenerative diseases. Cellular senescence is a state of irreversible cell cycle arrest characterized by distinct morphological, functional, and molecular alterations. Experimental evidence from pharmacological elimination of senescent cells (SCs), SC transplantation, and transgenic mouse models has established a causal link between SC accumulation and age-associated tissue dysfunction (Singh and Singh 2025). Notably, the introduction of SCs has been shown to accelerate aging-related phenotypes, whereas their targeted removal can ameliorate these effects. In the aging brain, the presence of SCs increases significantly in murine models and is correlated with cognitive deterioration. Importantly, senescent cell ablation not only reduces neuroinflammation but also markedly delays or even prevents cognitive impairment (Shafqat et al. 2023).

Senescence is linked to autophagy and influences organ aging beyond chronological age. The long-term effects of intermittent fasting on autophagy, inflammasome activity, and senescence remain unclear (Erlangga et al. 2023). The effects of aging might initially be subtle, manifesting as a slight decrease in stamina or a forgettable moment. However, as we age, this decline becomes more pronounced. Unfortunately, the risk of succumbing to various diseases increases with age. This inherent vulnerability necessitates a focus on preventative healthcare and early detection strategies. Additionally, as we age, cellular metabolism, how our cells utilize energy, undergoes alterations (Cohen et al. 2022). This shift may profoundly influence health and longevity. Knowing the complex nature of aging enables us to address it proactively. By embracing healthy living habits and the application of medical technologies, we can work toward enhancing our health span, or the number of years lived in the absence of chronic disease (Fairweather 1991).

This review offers a broad overview of the process of aging, characteristic features, and the process of IF-induced activation of healthy aging by inducing autophagy. The cellular and molecular machinery of aging includes telomere shortening, epigenetic modification, oxidative stress, mitochondrial dysfunction, and cellular waste accumulation. In addition, the advantages of IF-induced autophagy to cognition and neurodegenerative disease will be described, as well as its role in lifestyle- and health-related disorders.

## Aging and its Hallmarks

Aging is a multifaceted, temporally evolving biological process controlled by a cascade of molecular, environmental, and lifestyle determinants that lead to loss of physical and cognitive function (Amarya et al., 2018; Jin, 2010). A recent study has divided these hallmarks into three categories: primary, antagonistic, and integrative (Tartiere et al. 2024). The primary hallmarks of aging include genomic instability, telomere attrition, epigenetic alteration, and loss of proteostasis. Genomic instability comprises all accumulated damage in the genome, either arising from mutations or chromosomal alterations over time. On the other hand, epigenetic alterations refer to changes in the genome caused by environmental factors, without changes in DNA sequence. Telomere attrition refers to the shortening of telomeres during subsequent replication, which limits the maximum capacity for replication. Lastly, loss of proteostasis refers to an imbalance in protein synthesis, functioning, and degradation (Tenchov et al. 2024).

Antagonistic characteristics of aging include deregulated nutrient sensing and macroautophagy, mitochondrial dysfunction, and cellular senescence. Deregulated nutrient sensing occurs due to maladaptive changes in cellular sensing and response to nutrient availability, and an impairment of cellular pathways that detect the availability of nutrients (López-Otín et al. 2013). Impaired mitochondrial function is the age-related decline in mitochondrial function and decreased efficiency in mitochondrial quality control, leading to the accumulation of dysfunctional mitochondria and ROS. Oxidative stress, caused by an imbalance between ROS generation and antioxidant defenses, is a leading cause of macromolecular damage and cell dysfunction. Increased ROS levels and mitochondrial dysfunction can cause mitochondrial membrane permeabilization, thus triggering inflammation and cell death (Li et al. 2023).

Cellular senescence is one of the most prominent characteristics of biological aging, wherein aged cells become more prone to senescence owing to disturbances in cellular homeostasis resulting from aging (Tartiere et al. 2024). Aging is a factor that induces DNA damage, telomere shortening, and oxidative stress, thereby triggering the DNA damage response as well as activation of p53. The activated protein, in turn, stimulates the production of cyclin-dependent kinase inhibitors, such as p21 and p16, resulting in blockage of CDK-cyclin complexes and resulting in a halt of cell cycle progression. Mitochondrial dysfunction and an increase in reactive oxygen species also enhance senescence via DNA damage and activation of p53 by p38 MAPK (Ajoolabady et al. 2025).

## Current Hallmarks of Brain Aging

Brain aging is also marked by a gradual reduction in cognitive and motor abilities, which is reflected by changes in the structure of the brain. Normal aging is also a condition because it is accompanied by a decline in the volume of gray and white matter, as well as regression of the dendrites and a loss of neurons, which is also reflected in a decline in the abilities of learning, memory, attention, and perception (Mattson and Arumugam 2018).

At the cellular/molecular level, aging has been found to deeply influence the brain’s numerous homeostatic and stress response systems. Features of brain aging include mitochondrial dysfunction with ATP depletion and increased oxidative damage to proteins, lipids, and nucleic acids, disruption of intracellular waste removal mechanisms like autophagy and proteasome systems, disruption of DNA repair machinery, altered neuronal calcium regulation, and dysfunctional stress response systems (Wilson et al. 2023). These changes are accompanied by dysfunctional activity within neuronal circuits and the presence of chronic inflammation, which have contributed to reduced neuronal durability in the brain due to aging (Mattson and Arumugam 2018; Gaspar-Silva et al. 2023).

Moreover, certain environmental and lifestyle variables further intensify the described biological processes, where metabolic conditions that disrupt bioenergetic balance, like exercise and intermittent energy restriction, may increase stress resilience and protect against functional decline, while overeating and a sedentary lifestyle accelerate the pathophysiology of brain aging (Gaspar-Silva et al. 2023).

## Age-Related Changes in Brain Structure

The first major change in the brain as we age is a decrease in brain volume or mass. Lifestyle choices, such as diet, physical activity, and cognitive engagement, are among the major factors that influence brain aging and potentially mitigate the effects of shrinkage (Peters 2006). Regions of the brain with high metabolic activity and sensitivity to oxidative stress, including the frontal lobe and hippocampus, have the greatest degree of shrinkage with age. The susceptibility of the aged population is further increased by their high receptor density for neurotransmitters and hormones, which decrease with age. Decreased blood flow and increased white matter lesions also occur disproportionately in these regions. All these factors contribute to the structural changes observed in the frontal lobe and hippocampus (Raz et al. 2007; Fabiani et al. 2015). Cortical thickness decreases with age, especially in the frontal and temporal lobes. Thinning usually begins in middle age, and this condition is most likely associated with a loss of both synaptic connections and neuronal cell bodies, resulting in lower cortical density and potentially contributing to slower cognitive processing (Salat 2004).

During aging, gray matter volume shrinks across the board, with the frontal and temporal lobes, crucial for thinking and memory, taking the greatest hit. This decline is not uniform; however, some regions lose volume faster in nonlinear patterns. Substantial GM volume reductions are also observed in the parietal lobe, whereas the occipital lobe appears relatively intact. Recent studies have documented a decline in GM volume in the cerebellum, which includes areas associated with cognitive functions (Ramanoël et al. 2018). Interestingly, women tend to lose gray matter in the frontal lobe at a slower rate than men do. These regional differences, and possible sex differences, underscore the complexity of brain aging and its influence on cognitive function. The white matter undergoes a series of changes during the process of aging. It is essential for the transmission of electrical signals across different brain regions, and white matter malfunction can therefore lead to severe neurobehavioral and cognitive impairments (Liu et al. 2017). Additionally, regional variations exist, with the frontal and temporal lobes exhibiting more pronounced volume loss than other areas (Salat et al. 2009). Diffusion tensor imaging (DTI) reveals microstructural changes with aging, as evidenced by alterations in fractional anisotropy (FA) and mean diffusivity (MD), potentially indicating that white matter integrity is compromised. There are even sex differences, and it could be the case that, particularly in certain areas, the decline in white matter volume is potentially at a slower rate in women. These results highlight the complexity of age-related white matter changes (Kaļva et al. 2023).

## Age-Related Changes in Brain Function

Memory encoding and retrieval engage multiple neural circuits, and these engagement patterns shift with advancing age. Initial investigations proposed that, compared with their younger counterparts, older adults exhibit reduced neuronal engagement and involve alternative brain regions. However, those early observations did not indicate that younger individuals typically achieve higher task accuracy, which could influence neural activity regardless of age (Deery et al. 2023). Subsequent studies addressed this issue by categorizing older adults based on their memory task outcomes and comparing their brain responses to those of younger participants. The results revealed that older adults with strong performance presented brain activity patterns comparable to those of younger individuals, as measured through blood oxygen level–dependent signal imaging. This suggests that the quality of neural activation is more indicative of task success than chronological age and that aging may lead to reduced activation specificity.

An associated explanation for these changes in neural dynamics is known as the posterior-to-anterior shift in later life. This model proposes that, with age, there is a shift toward activating frontally located brain areas, including the prefrontal cortex, to offset deficits in rear brain regions. A recent investigation used a model-driven multivariate technique to assess whether heightened activity in the prefrontal cortex in older adults served a compensatory function or reflected ineffective engagement. The findings indicated that elevated prefrontal signals corresponded with decreased specificity and lower cognitive efficiency. Comparable patterns have been noted in aged animal models; for example, older rodents with memory impairments exhibit irregular activation in both cortical and hippocampal subregions. As in humans, this exaggerated activation was absent in older rodents with preserved cognition, implying a diffuse or non-targeted recruitment response (Morcom and Henson 2018).

In addition to the hippocampus and cortical areas, regions involved in movement control are also impacted by the aging process. Research has shown that following motor skill learning, elderly individuals exhibit greater activity in multiple brain cortex areas, as well as in the cerebellum, as compared to younger individuals (Zapparoli et al. 2022). A more recent study assessing neural responses during spatial orientation tasks reported heightened activation in the cerebellum of older individuals (Tragantzopoulou and Giannouli 2024). In contrast, separate findings have shown diminished cerebellar involvement during motor learning in the same age group. These opposing outcomes suggest that cerebellar function may shift with age. Collectively, these results imply that the aging process influences activity across multiple brain regions, including the cerebral cortex, hippocampal areas, and cerebellum, although further research is necessary to validate how specific tasks are uniquely affected (Hardwick and Celnik 2014).

**Fig. 1:**
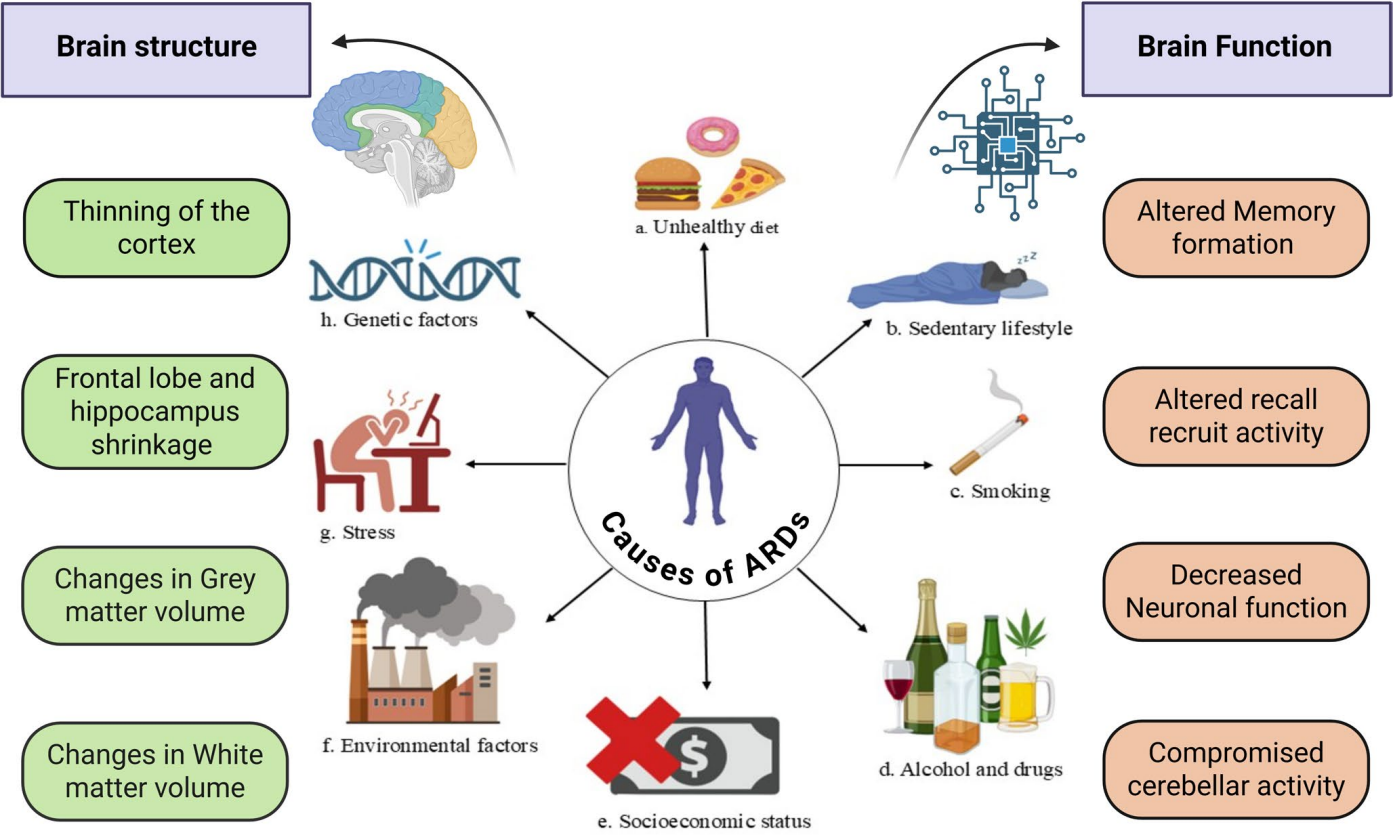
Age-associated disorders can be caused by multiple factors such as consumption of an unhealthy diet, sedentary lifestyle, alcohol and drug abuse, environmental and genetic factors, as well as stress. This consequently results in changes in brain structure and function, including thinning of the cortex, changes in grey and white matter volume, hippocampus shrinkage, altered memory and recall recruit activity leading to reduced neuronal function

Changes in mental capabilities with increasing age are extensively documented. Not all cognitive domains are influenced equally: some remain relatively stable or even strengthen, whereas others exhibit a gradual reduction (Zihl and Reppermund 2023). For instance, language-related knowledge often remains intact and may continue to expand. In contrast, abilities such as abstract thinking, rapid information handling, and memory retrieval tend to deteriorate over time. Although these cognitive adjustments are a natural part of aging, the extent and speed of these adjustments vary between individuals. Faster decline is frequently associated with a reduced capacity for daily functioning and the onset of neurodegenerative conditions, such as Alzheimer’s disease and cerebrovascular dementia, the two most prevalent cognitive disorders in elderly individuals (Gómez-Gómez and Zapico 2019). Furthermore, variability in cognitive trajectories is observed not only in humans but also in experimental animal models. This diversity leads to the classification of aged individuals or animals as either high or low cognitive performers, emphasizing the need for precise behavioral tools capable of effectively differentiating between them **(**Fig. 1**)**.

## Cellular Mechanisms Involved in Brain Aging

In terms of brain cellular aging, the process involves a complex scenario involving neuronal loss and compensation with the expansion of dendritic arbor and synaptic connections. The degree of compensation does not readily restore neuronal loss, which leads to a decline in synaptic density, with inefficient communication between the neurons (Zia et al. 2021).

Dendritic regression, or thinning of dendritic branches, reduces the capacity of the brain to process information as a result of the fundamental role played by these structures in the integration and transmission of neural messages (Luiten et al. 2020). Another problem is the cytoskeleton, a fundamental cellular morphology component that bestows structural integrity on the cell. The aging brain correlates with cytoskeletal abnormalities that include microtubule instability and disruption of actin filaments; these may hamper cell function and contribute to the development of neurodegenerative disorders. The impaired cognitive processes of aging are established by various cellular and molecular changes, gene expression changes, oxidative stress, and inflammatory processes (Zia et al. 2021).

Cellular mechanisms of brain aging are a sequence of linked events causing progressive neuronal dysfunction and disruption of brain homeostasis. Oxidative stress lies at the heart of the process, which originates from the imbalance of reactive oxygen species (ROS) due to mitochondrial dysfunction. With the aging of the organism, the efficiency of mitochondria in energy generation decreases in parallel with the production of an increased amount of ROS, thus initiating oxidative damage to proteins, lipids, and DNA.

Neurons are especially vulnerable to oxidative stress due to their postmitotic state and high energy requirement. In addition, DNA damage and genomic instability accumulate with aging due to the loss of efficacy of repair mechanisms, i.e., base excision repair (BER) and nonhomologous end joining (NHEJ). These genomic changes lead to gene expression and neuron viability disruptions, and eventually neurodegeneration. The second main factor is the perturbation of proteostasis, which encompasses the cellular processes of protein folding and degradation. Aging compromises the effectiveness of autophagy and that of the ubiquitin–proteasome pathway, leading to the deposition of misfolded or aggregated proteins. Accumulation of these misfolded proteins is neurotoxic and is a hallmark of numerous neurodegenerative disorders. Senescence in glial cells also leads to the secretion of proinflammatory mediators known as the senescence-associated secretory phenotype (SASP). The mediators stimulate microglia and astrocytes, hence inducing chronic neuroinflammation that eventually harms neurons and synapses. All these events are an insidious interplay of cellular degeneration that leads to cognitive impairment and structural brain aging.

## Age-Related Lifestyle Diseases and Preventive Measures

Age-related lifestyle diseases (ARDs) are chronic conditions that become more frequent with advancing age, typically compounded by individual lifestyle and environmental determinants. ARDs are mostly non-communicable and include a wide range of medical disorders. With progressive chronological aging, the prevalence of many pathologies steeply rises, all of which fall under the category of age-related disorders or senescence-accelerated diseases. Prevention of age-related lifestyle diseases requires an integrated approach to manage the causative risk factors involved in health maintenance. Introduction of modifications in lifestyle, dietary interventions, and pharmacotherapy are essential to counter the adverse effects of aging on the health of individuals.

### Lifestyle Modifications and Relevance to ARD

Lifestyle modification can enhance the quality of life, thereby significantly decreasing the risk of age-related diseases (ARDs). The integration of regular physical activity is essential in the prevention of the risks of cognitive impairment and cardiovascular diseases (Kivipelto et al. 2018). A function-based preventive restriction targeting physically inactive individuals demonstrated that structured physical activity can significantly improve musculoskeletal health and overall fitness (Larsen et al. 2018). Also, regular exercise is associated with fewer chronic diseases and thus improves health-related quality of life, which includes cardiovascular disease and metabolic disorders (Amiri et al. 2024). Lifestyle modification can be geared toward the prevention of the development of Alzheimer’s disease; however, interventions for the improvement of awareness of the need to maintain cognitive stimulation and social interaction have shown that cognitive decline and impairment can be reversed (Ko and Chye 2020). Also, interventions such as “My Healthy Brain” have shown efficacy in the integration of lifestyle modification in healthcare settings, overcoming obstacles in their delivery (Mace et al. 2024). Sweeping modification of diet is essential in preventing ARDs. For example, the increased intake of low-fat dairy foods and fish improves calcium and vitamin D levels, which are crucial in the maintenance of bone integrity (Everitt et al. 2006). A fruit- and vegetable-rich diet provides antioxidants and anti-inflammatory compounds, which are essential in the prevention of neurodegenerative disease (Román et al. 2019). The intake of whole grains over refined grains decreases the risk of cancer development and improves overall well-being (Riccardi et al., 2022). Keeping to a regular and time-based eating regimen improves the regulation of metabolism and prevents the development of hypertension (Jamshed et al. 2019). Besides, accepting intellectual challenges, such as acquiring new skills or engaging in complicated tasks, is essential in ensuring cognitive function and preventing decline (Mattson 2020). In addition, teaching patients to accept healthier lifestyles empowers them with better life habits (Beyer et al. 2024). Lifestyle Medicine Centers can also provide formal programs to put such constraints in place, thus eliminating issues like the social stigma of perception and limited resources (Mattson 2020; Mace et al. 2024).

### Pharmacological Strategies for Prevention of ARDs

Recently, new pharmacological strategies for addressing age-related lifestyle disorders have increasingly turned to interventions that target the mechanistic pathways of aging. Various research avenues indicate promising methodologies, including drug delivery systems and targeted molecular agents. One strategy against the prevention of age-related disorders (ARDs) is anti-inflammatory therapy via the inhibition of the interleukin-6 pathway, which has been shown to relieve many ARDs, including diabetes and cardiovascular diseases (Qu et al. 2014; Bowker et al. 2020). Further, molecules like metformin and rapamycin have been investigated for their ability to extend health span and reverse ARDs (Li et al. 2021). Compounds like rapamycin and metformin are potentially effective in slowing the aging process and preventing diseases related to age as studied in both humans and animal models by inhibiting the mTOR pathway, which is linked to reduced cellular senescence, enhanced metabolic health, and reduced risks for age-related disorders, including cancer and cardiovascular disease (Aliper et al. 2017). Advanced DDSs have the potential to address age-related lifestyle disorders by enhancing bioavailability and targeting therapeutics, thereby enhancing drug efficacy while minimizing side effects, such as nanoparticles that can deliver anti-inflammatory therapeutics to senescent cells, which effectively suppress inflammatory responses linked to aging (Yoshihara and Horiguchi 2024).

## Intermittent Fasting (IF) and its Implication in Senescence

IF and caloric restriction are two different dietary regimens that have been demonstrated to improve several metabolic parameters, among them the control of body weight. CR consisted of reducing overall daily energy intake while continuing to provide nutritional requirements. The IF, on the other hand, cycled between food deprivation and scheduled eating windows. Studies in human and animal models indicate that the benefits of IF go far beyond fat reduction and reduced oxidative stress. IF can trigger conserved mechanisms of biology that regulate interorgan communication, improve glucose control, increase resistance to physiological stress, and downregulate inflammatory processes. Cellular mechanisms during fasting increase protection against oxidative and metabolic stress and become more proficient at sensing and repairing or clearing defective components. In addition, these processes have tissue-specific roles in mediating neural adaptability and growth, which become activated during eating windows. Triglycerides deposited in adipose tissue are broken down to glycerol and fatty acids, which are used as a source of fuel during food-free intervals. The liver metabolizes these fatty acids to ketones, an essential source of fuel for several organs, including the brain. In humans, circulating ketones are low following meals but start to increase within 8–12 h of food deprivation and accumulate to concentrations of around 2–5 millimolar at the 24-hour time point. In rodents, blood concentrations of ketones start to increase between 4 and 8 h after the onset of fasting and can reach 1 millimolar after one day of food withholding.

The timing of physiological responses offers guidance on ideal fasting durations in IF strategies. The most studied IF methods in humans include alternate-day fasting, the 5:2 plan (fast 2 days weekly), and daily time-restricted eating. Ketone levels rise when caloric intake is sharply reduced on fasting days. This shift from glucose to fat and ketone metabolism lowers the respiratory exchange ratio, indicating increased metabolic flexibility and energy efficiency. Therefore, dietary patterns should consider not only the amount and type of food consumed but also the frequency, timing, and duration of fasting. IF focuses on when food is consumed rather than how much or what kind of food is consumed **(**Table 1**)**.

**Table 1:** List of various types of intermittent fasting, their methodologies, benefits, risks, and associated case studies

Study	Model/Population	IF Protocol & Method	Key Findings	Reference
Effects of intermittent fasting on brain aging & cognition in older insulin-resistant adults	Older adults (≈ 40)	5:2 IF (2 low-calorie days/week, 8 weeks)	IF improved executive function, reduced brain-age gap (MRI), and improved metabolic biomarkers; minimal changes in Alzheimer’s CSF biomarkers.	(Kapogiannis et al. 2024)
Prolonged nightly fasting & cognitive function in older adults with memory decline	Older adults (≥ 65, *n* ≈ 18)	14-h night fasting for 8 weeks	Increased cognitive function (memory/attention) and reduced insomnia severity, promising pilot evidence.	(James et al. 2025)
Systematic review: IF, caloric restriction & BDNF/Cognition in humans	Humans (multiple studies)	Reviewed ADF, CR, TRE, RIF	Mixed effects on BDNF; some IF protocols improved cognition, others did not; evidence is heterogeneous.	(Alkurd et al. 2024)
Intermittent fasting does not affect adult hippocampal neurogenesis in mice (5:2)	Mice (adult & adolescent)	5:2 IF for 6 weeks	5:2 IF did not increase adult hippocampal neurogenesis nor enhance spatial learning.	(Roberts et al. 2023)
IF alleviates postoperative cognitive dysfunction in aged mice	Aged mice	Pre-surgery IF (≥ 2 weeks)	IF reduced neuroinflammation, increased synaptic protein expression, and improved cognition after surgery.	(Madorsky et al. 2009)
IF protects against radiation-induced brain damage (rat model)	Rats (radiation model)	ADF/TRF variants	IF reduced brain damage via IRS-1/PI3K/AKT and BDNF/TrkB signaling.	(Taha et al. 2025)
IF modulates neurotransmitters and oxidative stress in the rat brain	Rats	24-h IF for 1–15 days	IF altered neurotransmitter levels and oxidative stress markers; short-term increases in BDNF in hippocampus; complex temporal effects.	(Abdel-Rahman et al. 2024)
Orexin-A & BDNF changes with IF + physical activity (middle-aged rats)	Middle-aged rats	IF ± voluntary activity (8 weeks)	IF elevated hippocampal Orexin A & BDNF, combined activity amplified effects and motor behavior.	(Ebrahimi et al. 2025)
Systematic review: TRE & IF on cognition & mental health in older adults	Older adults (n studies)	TRE/IFA	TRE/IF is regularly associated with cognitive/mental health benefits in some studies; mixed quality.	(Sharifi et al. 2024)
IF alters gut microbiota & ameliorates cognitive decline in AD mice	AD mouse model (5XFAD)	IF regimen (model-specific)	IF improved cognition, reduced Aβ burden, and altered gut microbiota metabolites.	(Pan et al. 2022)
Traditional IF enhances hippocampal neurogenesis & longevity gene expression (mouse)	Mice	Every other day, IF (3 months)	IF increased neurogenesis, BDNF, and expression of longevity gene *Klotho* vs. CR and ad libitum.	(Dias et al. 2021)
Systematic review of multiple RCT dietary interventions on cognition	Adults (multiple RCTs)	Diet/IF reviewed	Suggestive evidence that dietary interventions, including IF, may support cognition but the evidence is limited/low quality.	(Senderovich et al. 2023)
Emerging clinical evidence: IF & neurocognitive disorders review	Animal & human evidence	Review	IF enhances hippocampal plasticity and reduces inflammation in preclinical models, limited translational evidence.	(Beveridge et al. 2025)

IF has been linked to extended lifespan and improved health through enhanced autophagy and reduced inflammation. The following table describes the various studies incorporating different IF interventions in clinical testing, summarizing their study parameters and primary outcomes (Table 2**)**.

**Table 2:** Various IF interventions and their outcomes in human studies

IF Intervention	Description	Primary Outcomes in Human Studies	References
Continuous Energy Restriction (CER)	Daily caloric intake reduced by ~ 20–30% below energy requirements without fasting periods. Often used as standard comparator in IF trials.	Consistent weight loss; improvements in BMI, lipid profile, insulin sensitivity. Comparable efficacy to IF when caloric deficit is matched.	(Harvie et al. 2013; Welton et al. 2020)
Time-Restricted Eating (TRE)	All caloric intake confined to a daily eating window (e.g., 16:8, 14:10, 12:12), without intentional calorie restriction.	Moderate weight loss; reduced fat mass and waist circumference; variable effects on glucose and lipids; improved insulin sensitivity in some cohorts.	(Wilkinson et al. 2020; Cienfuegos et al. 2020, pp. 4-; Lowe et al. 2020)
Alternate-Day Fasting (ADF)	Alternating 24-h fasting days with ad libitum feeding days; modified ADF allows ~ 25% energy intake on fasting days.	Significant reductions in body weight, fat mass, LDL-C, and triglycerides, often superior to TRE; improvements in insulin resistance.	(Varady et al. 2009; Cai et al. 2019)
5:2 Intermittent Fasting Diet	Severe caloric restriction (~ 500–600 kcal) on two non-consecutive days per week; normal intake on the remaining five days.	Weight loss comparable to CER; improved insulin sensitivity; reduced hepatic steatosis and fibrosis in metabolic liver disease.	(Harvie et al. 2011; Carter et al. 2018)
Whole-Day Fasting (Periodic Fasting)	Complete fasting for 24–48 h at regular intervals (weekly or biweekly), followed by normal eating.	Reduction in body weight and cardiometabolic risk; limited long-term RCT data; greater metabolic switching.	(Liu et al. 2022)
Fasting-Mimicking Diet (FMD)	Short-term (3–5 days/month) very-low-calorie, low-protein, low-carbohydrate diet designed to mimic fasting physiology.	Reduced IGF-1, glucose, triglycerides, and CRP; improved metabolic and inflammatory markers.	(Brandhorst et al. 2015; Wei et al. 2017)
Intermittent Fasting Meal Replacement (IF-MR)	IF combined with meal replacements on fasting days (often 5:2 format).	Greater HbA1c reduction, weight loss, and insulin sensitivity in type-2 diabetes patients compared to standard care.	(Guo et al. 2024)
Extended Fasting (> 48 h)	Prolonged fasting lasting 48–72 h or longer; mostly studied mechanistically rather than clinically.	Ketosis, metabolic switching, and autophagy activation; limited controlled human trials; safety concerns in long-term use.	(Mattson et al. 2017)
5:2 time-restricted eating regimen	It compared the impacts of a 5:2 time-restricted eating regimen and a standard healthy diet on neurological health.	Fasting protocol led to more significant body mass reduction, similar outcomes in enhancing insulin-related markers found in neuron-origin extracellular particles, narrowing the brain-age difference, and lowering cerebral glucose levels. Improved sugar and fat metabolism. Enhanced cognitive abilities.	(Kapogiannis et al. 2024)

## Autophagy and Maintenance of Cellular Homeostasis

The conserved regulatory pathway termed autophagy plays a significant role in maintaining homeostasis and responding to stress. This pathway encapsulates fragments of the cytoplasm, such as organelles and proteins, in double-membrane vesicles called autophagosomes. Upon autophagosomes fusing with lysosomes, the components undergo breakdown by hydrolytic enzymes, resulting in macromolecules re-circulating in metabolic processes (Aman et al. 2021). The molecular mechanisms of autophagy can broadly be classified into several important phases that are important to its overall process.

This degradation pathway is initiated, resulting in the formation of a phagophore that elongates to engulf cellular components (Majeed et al. 2022). As the phagophore elongates, it eventually closes, thus forming an autophagosome, and it later fuses with lysosomes to form an autolysosome. In the autolysosome, hydrolytic enzymes catalyze the breakdown of its contents into simple macromolecules that are secreted back into the cytoplasm to be reused (Lu et al. 2022).

This degradative pathway is tightly regulated by several linking mechanisms, including autophagy-related (ATG) proteins that are central regulators of various stages of autophagy; the mechanistic target of rapamycin (mTOR) pathway, a master suppressor of autophagy under rich nutrient conditions; conversely, AMPK activation in nutrient depletion (Yang and Klionsky 2009).

**Fig. 2:**
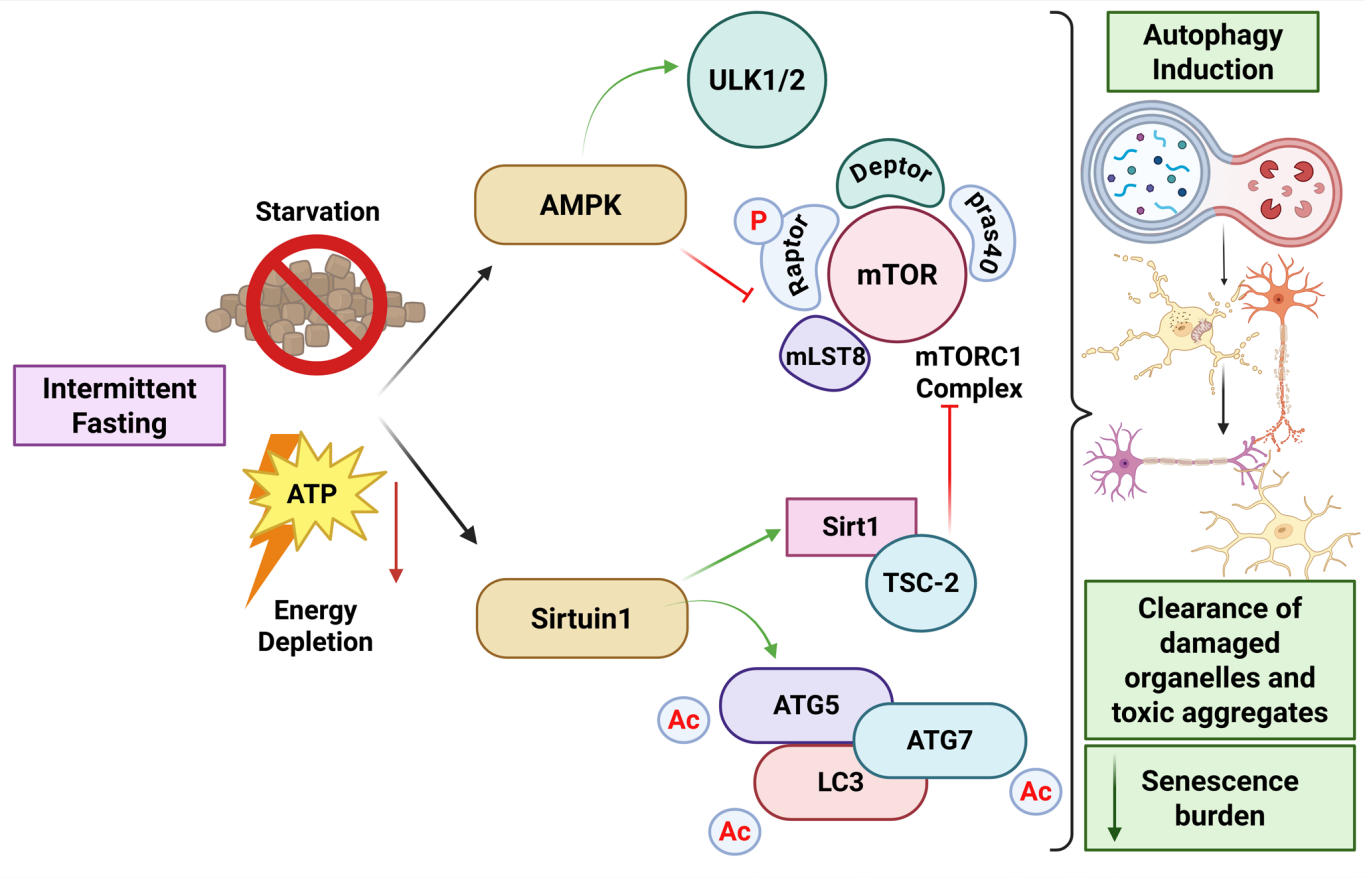
IF is associated with energy deprivation, such as conditions of starvation, that facilitate the regulation of AMPK and Sirtuin1 pathways. Upregulation of AMPK pathways leads to activation of ULK1/2 as well as inhibition of the mTORC1 complex. On the other hand, the upregulation of the Sirtuin1 pathway helps in the deacetylation of multiple autophagy-related genes such as ATG5 and ATG7, as well as LC3. Activation of Sirt1 also leads to inhibition of the mTORC1 complex. As a result of this cascade, the process of autophagy is activated, leading to the clearance of senescent cells

Autophagy is pivotal in the preservation of cell integrity by eliminating defective organelles and misfolded proteins, maintaining osmoregulation by recycling cell material for energy, and contributing to immune function. Thus, dysregulation is linked to various diseases, including cancer and neurodegenerative diseases. Therefore, a comprehensive understanding of the molecular mechanisms governing autophagy not only elucidates its fundamental biological roles but also unveils promising avenues for therapeutic intervention in a broad spectrum of pathological conditions (Jiang et al. 2019; Cui et al. 2019) (Fig. 2).

The role of selective autophagy has been identified as an important factor in determining neuronal fate during neurodegenerative conditions. The malfunctioning of mitophagy has been shown to directly contribute to Parkinson’s disease; in other words, mutations in PINK1 and Parkin have been shown to impair mitophagy and thereby contribute to Parkinson’s disease by causing an abundance of dysfunctional mitochondria along with increased levels of oxidative stress and resulting in degeneration of dopaminergic neurons in cellular models and MHD mice (Pickrell and Youle 2015). The malfunctioning of mitophagy has also been shown to contribute to impairments in synaptic functions, along with impairments in memory in animal models of AD; in other words, improving mitophagy has been shown to improve amyloid plaques and tauopathy, along with memory functions in AD animal models. Moreover, dysfunctional pexophagy has been shown to deprive maintenance of peroxisomes and has been implicated in lipid/homeostasis, along with oxidative stress in an aged brain; in other words, it has been shown to enhance conditions of degenerating neurons, along with inflammation in AD animal models (Nordgren and Fransen 2014).

## Role of Autophagy in Proteinopathy and Proteotoxicity

Disturbed proteostasis, proteinopathy, and proteotoxicity are somehow connected, as they influence cellular health, at least in neurodegenerative conditions. In this context, autophagy participates in maintaining proteostasis by degrading aberrantly folded protein aggregates before their accumulation causes cell death and toxicity through misfolded protein assemblies, such as those in Alzheimer’s disease and Parkinson’s disease (Lam et al. 2020). This involves improper proteostasis, a failure of protein quality control mechanisms contributing to the accumulation of toxic proteins and neurodegenerative diseases (Tseng et al. 2023). In the CNS, autophagy is an essential mechanism maintaining the proper function of post-mitotic cells, including neurons and glia. Loss of core autophagy genes in neuronal populations leads to neurodegeneration in animal models, underscoring that basal autophagic flux is absolutely required even in the absence of overt metabolic stress (Griffey and Yamamoto 2022). Microglial autophagy contributes to synaptic pruning, the clearance of extracellular amyloid-β, and the regulation of innate immune responses, while astrocytic autophagy participates in the degradation of misfolded proteins and the modulation of inflammatory signaling, with evidence that enhancing astrocytic autophagy promotes the clearance of pathogenic aggregates and supports neuronal integrity. Dysfunctional autophagy in these glial populations indeed promotes impaired neuroprotection, increased inflammation, and neurotoxic substrate accumulation, thereby linking altered autophagy status to aging and neurodegenerative processes (Griffey and Yamamoto 2022).

In Drosophila models of Alzheimer’s disease, increased autophagic activity is protective against protein aggregation (Ortiz-Vega et al. 2024). The sigma-1 receptor is a critical factor that links autophagy and proteostasis: its activation enhances autophagic processes that counteract neurodegeneration (Christ et al. 2020). Targeting autophagy pathways offers a promising therapeutic strategy to restore proteostasis and combat proteinopathy effects, particularly in metabolic cardiomyopathy and neurodegenerative conditions.

While the interplay between disturbed proteostasis, proteinopathy, and autophagy is critical for understanding neurodegeneration, it is also valuable to recognize that not all autophagic responses are favorable; overactivation can result in cell death and, thus, the need for balanced regulation in therapeutic approaches.

## Effects of IF-induced Autophagy on Brain Health and Neuroprotection

Intermittent fasting (IF) is rapidly being acknowledged as a neuroprotective strategy, with accumulating evidence supporting the fact that the neuroprotective effects of IF are at least in part countered through the augmentation of autophagic flux. IF causes the switching on of metabolism from the usage of glucose to the usage of fatty acids and ketones, resulting in the downregulation of mTORC1 and the stimulation of AMPK, two pivotal regulators of macroautophagy (Longo and Mattson 2014). The activation of autophagy induced by fasting promotes the efficient breakdown of damaged organelles, aberrantly folded proteins, and toxic protein aggregates through lysosomal degradation, which is a critical phenomenon for the sustenance of neuronal homeostasis in the-aged brain (Mattson et al. 2017).

Maintaining the overall balance of autophagy might fight against the adverse effects of decreasing bodily function with age. Individuals might further develop robust immunity while also experiencing a decrease in disease-causing chronic risk factors. Sticking to regular, well-balanced diets coupled with healthy working exercises in the pattern of short-term periods of abstinence during meals is how people today can achieve more positive thinking toward combating their aging cycles. Fasting enhances autophagy, a vital intracellular mechanism in neurons of the cerebral cortex and cerebellum. This metabolic state may trigger the degradation of internal cell materials to preserve energy balance, supporting neuronal survival under nutrient-limited conditions. It also influences mTOR signaling, leading to enhanced autophagic activity. Persistent alterations in synaptic efficiency are viewed as necessary for cognitive processes, including memory formation, and the mechanistic target of rapamycin (mTOR) plays a central role in controlling local protein synthesis at synapses in dendrites and is implicated in various forms of synaptic plasticity. IF is recognized as a potent autophagy inducer for homeostasis and cellular integrity. It triggers a cascade of molecular signaling, increasing autophagic activity, particularly in the liver, a major organ of metabolic control and disease prevention, acting through AMP-activated protein kinase (AMPK) activation, mTOR inhibition, and SIRT1 modulation (Ma et al. 2023). From several studies, IF enhances hepatic autophagy, which enhances liver function and offers therapeutic benefits against nonalcoholic fatty liver disease (NAFLD) (Hosny et al. 2023; Kim et al. 2023). Further, IF inhibits hepatic lipid accumulation and inflammation by the autophagy-lysosomal pathway, which is a major component in steatohepatitis with metabolic derangement (Kim et al. 2023).

mTOR, a protein kinase, is activated by oxidative stress through the phosphoinositide 3-kinase/protein kinase B (PI3K/Akt) pathway, leading to cellular growth and release of inflammatory cytokines (Roy et al. 2023). Also, Fasting- or exercise-mediated energy stress induces AMPK, inhibits mTOR activity in muscle cells (Mounier et al., 2015). Furthermore, AMPK activation in the hippocampus is involved in brain adaptation, as in exercise-conditioned mice, and is correlated with improved memory (Marosi et al. 2012).

**Fig. 3:**
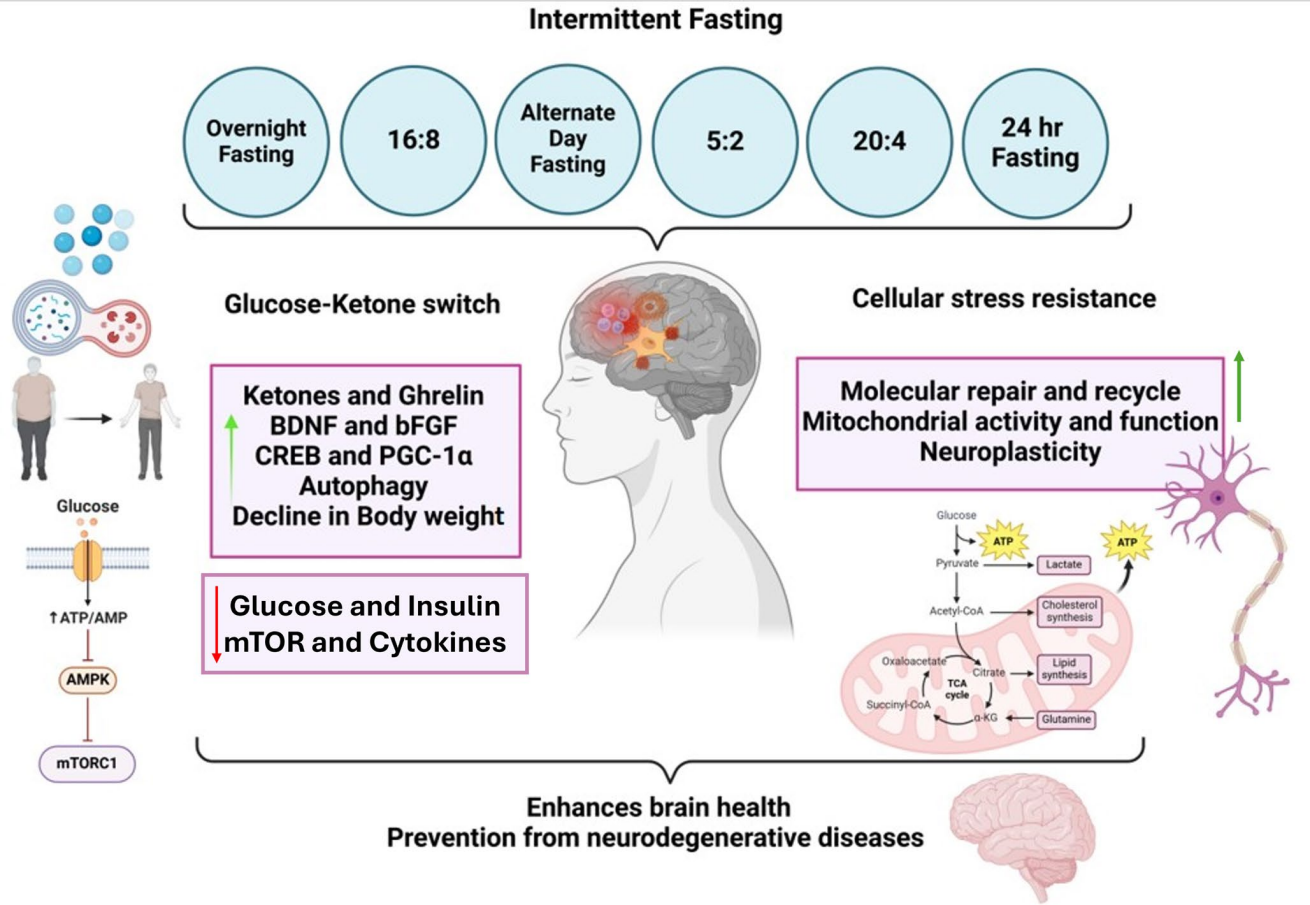
IF is a dietary restriction alternating between periods of abstinence and timed eating phases. It can activate conserved biological mechanisms that coordinate interorgan communication, enhance glucose regulation, improve resistance to physiological stress, and diminish inflammatory responses. IF has been linked to extended lifespan and improved health through enhanced autophagy and reduced inflammation

Energy sensor AMPK activates autophagy via inhibiting the central suppressor mTOR (Ma et al. 2023). SIRT1 and PPARα also upregulate autophagy by regulating lipid metabolism and autophagy-related cellular stress response genes. In addition, RUBCN degradation in adipocytes induces autophagy to allow lipids mobilization and ketone synthesis during fasting (Yamamuro et al. 2022). Also, during fasting, AMPK signaling is responsible for dendritic protrusions as well as for the potentiation of synaptic strength at the synapses of neurons in the arcuate nucleus of the hypothalamus. Rats treated with an AMPK-activating compound show improved learning capacity and improved body coordination, indicative of its contribution to inducing neuroplasticity (Kobilo et al. 2014). More importantly, transient activation of AMPK by brief metabolic stress, such as low cellular energy availability, can increase neural plasticity. However, chronic AMPK activation can disrupt the structural plasticity of axons and dendrites (Ramamurthy et al. 2014), which is by the necessity for recovery from fasting to achieve optimal neuroplasticity. While IF is beneficial for autophagy induction, excessive autophagy can have adverse effects, particularly in contexts such as chemotherapy, where it may exacerbate toxicity. Thus, the balance of autophagy modulation is crucial for therapeutic applications (Ozcan et al. 2023) **(**Fig. 3**)**.

 Neurons that function very significantly rely heavily on autophagic degradation for their performance and lifespan, which becomes essential (Nagayach and Wang 2024). Moreover, autophagy plays a critical role in managing oxidative stress by removing damaged mitochondria, which are sources of reactive oxygen species (ROS), thus preventing neurons from oxidative damage that causes neurodegenerative disease progression (Filomeni et al. 2015). Activated autophagy may have neuroprotective effects by modulating inflammation and promoting neuronal repair mechanisms, especially in inflammatory and neuronal loss diseases, such as Alzheimer’s disease (Cherra and Chu 2008). Considering the connection between defective autophagy and neurodegenerative diseases, enhancing such processes with dietary restrictions, for example, intermittent fasting, could be a promising therapeutic avenue (Li et al. 2024; Nagayach and Wang 2024).

Overall, IF-mediated upregulation of autophagic flux represents a central mechanistic link between metabolic adaptation and neuroprotection. By restoring autophagy-dependent proteostasis, mitigating mitochondrial dysfunction, and dampening chronic neuroinflammation, IF counteracts key hallmarks of brain aging and neurodegeneration, providing a strong biological rationale for its therapeutic potential in age-related neurological disorders (López-Otín et al. 2023).

It is, however, important to note that IF demonstrates neuroprotective functions through diverse mechanisms other than the activation of autophagy: overall metabolic dysregulation and regulation of central signaling pathways in brain cells. It increases overall metabolic health through the improvement of insulin sensitivity, decreases in glucose and insulin concentrations, and increased use of ketone bodies, which can secondarily affect brain function through the suppression of neuroinflammation and enhancement of insulin signaling in neurons (Mattson et al. 2018). On the other hand, it also triggers autophagy in other peripheral tissues, and the therapeutic effects on the brain could be attributed to both overall autophagic responses and the regulation of autophagy in specific brain cells. It is necessary to differentiate the overall effects of IF on autophagy and overall metabolic dysregulation and its effects on specific brain cells (Longo and Mattson 2014).

## Preclinical and Clinical Evidence

In a recent investigation, researchers reported that life expectancy has increased worldwide. Nevertheless, simply living longer does not ensure a healthier life. Age-related illnesses have become more prominent over the past few decades. Additionally, prior research has highlighted the advantages of intermittent fasting in delaying the onset of age-related diseases. Moreover, this approach is considered noninvasive and economical for preventing and treating various age-related diseases.

It was previously believed that fasting might not play a crucial role in the induction of autophagy in the brain. However, research conducted on C57BL/6 J and GFP-LC3 (Tg/+) mice, aged 6 to 7 months, has involved food withdrawal. Cellular recycling activity increased within 24 h and was even more pronounced after 48 h in liver cells, brain cortex neurons, and Purkinje neurons located in the cerebellum. A rise in the GFP-LC3 signal was observed, whereas phospho-S6RP levels in Purkinje neurons significantly decreased in response to food restriction. Since phospho-S6RP reflects the function of the mTOR pathway, its reduced presence suggests heightened autophagic activity, as mTOR signaling negatively regulates this process (Alirezaei et al. 2010).

A study was conducted to establish the effect of IF on autophagy in the management of age-associated disorders. Male candidates (*n* = 17) were recruited as a part of this study and were kept on IF for 17–19 h per day for 30 days. Blood samples were drawn from the candidates at four different time intervals: one week before the start of IF (TP1), two weeks after the start of IF (TP2), one month after the start of IF (TP3), and one week after the end of IF (TP4). These blood samples were then used to extract mRNA and assess the expression of genes associated with autophagy (ATG5, ULK1, and BECN1), inflammasome (NLRP3, IL-1β, ASC, and TNF-α), and senescence (p16INK4A, p21, and P53) through qPCR. Within two weeks of fasting, the ATG5, BECN1, and ULK1 levels increased compared with the basal levels. Prolonged intermittent fasting significantly induced ATG5 and ULK1 expressions but reduced the expression of another autophagy gene, BCN1. Prolonged intermittent fasting significantly induced the expression of the inflammasome genes NLRP3 and IL-1β but not its active component, ASC (adaptor molecule apoptosis-associated speck-like protein containing a CARD). The long intermittent fasting significantly reduced expression of p21, p16, and p53 at later times. The conclusion of the study showed that long IF regulated the activities of autophagy, inflammasome, and senescence with a time association. (Erlangga et al. 2023).

Another study was conducted to assess the effect of IF on the induction of macroautophagy in an Alzheimer’s disease model in vivo. Three-month-old male 5xFAD and control mice were used for in vivo analysis. To model Alzheimer’s disease, TAMRA-tagged human β-amyloid 1–42 (100 µM in ACSF, 0.1% DMSO; 1 µL) was injected into the right cortex under 1.5% isoflurane one day before imaging. The samples were fasted for 0 to 48 h (midnight day 1 to noon day 3), and water was provided freely. Weight and glucose were tracked during fasting. Time-lapse imaging revealed that food deprivation increased the number, size, and intensity of autophagosomes in neurons. These features were already elevated in Alzheimer’s disease model mice and rose faster than they did in controls during fasting. However, exogenous β-amyloid breakdown was limited, indicating that increased autophagy could not fully degrade the internalized peptide. Immunostaining confirmed that fasting increased internal β-amyloid buildup and cellular damage, with minimal changes in external deposits. These findings highlight altered neuronal autophagy dynamics in healthy and Alzheimer’s disease conditions (Chen et al. 2015).

Other preclinical evidence suggesting that IF is associated with autophagy came from a study in which C57BL/6J mice were subjected to IF (food was given on an alternative day). Following treatment, the sciatic nerves were isolated from both the control and IF groups. The results of this study revealed that intermittent fasting activated several internal defense processes in peripheral nerves, such as protein-folding support systems and cellular waste-clearance mechanisms. This intervention also reduced inflammation and halted degenerative features linked to neuropathy. Results show there was a decrease in the number of PMP22 clumps, as well as the presence of elevated levels of folding proteins in the cytosol, in conjunction with key molecules found in the process of autophagy, where there is an increase in the levels of Atg7 and LC3 (Madorsky et al. 2009).

The influence of IF on mental performance, especially in terms of its interaction with brain-derived neurotrophic factor (BDNF) and neural adaptability, remains largely unexplored in human trials. Nevertheless, new findings increasingly suggest that fasting strategies may support cognitive well-being. For example, earlier research demonstrated that, compared with caloric restriction (CR), time-limited eating protocols significantly enhanced mental function among overweight females while also contributing to greater fat reduction without negatively affecting appetite regulation, emotional state, rest patterns, or general wellness. Furthermore, a rigorously designed, forward-looking study conducted over three years involving 99 elderly Malaysian participants experiencing mild memory impairment who either engaged in consistent fasting, practiced it irregularly, or abstained altogether revealed that those who followed structured, prolonged fasting had better outcomes in all mental assessments than those who did not fast. Additionally, another early-stage study examined new neuron formation in the hippocampus, associated with learning ability, among centrally obese healthy adults. Participants following either a 5:2 eating schedule with unchanged caloric intake or a 25% reduction in dietary energy demonstrated measurable improvements in memory-based tasks, including pattern discrimination (Ooi et al. 2020)

IF can also lead to neuroprotective effects via the induction of autophagy in the case of Huntington’s disease (HD). HD is caused by the accumulation of a mutant form of huntingtin, which affects the loading of cargo into the autophagosome, thus destabilizing the autophagy process. They concluded that feeding at scheduled intervals is enough to upregulate SIRT1 and cause mTOR activation for the induction of autophagy in the YAC128 mouse model of HD (Ehrnhoefer et al. 2018).

## Risks and Considerations

The ideal age to begin intermittent fasting for reducing the risk of neurological conditions remains unclear, as most human research includes individuals across a broad age spectrum (Brandhorst et al. 2015; Kim et al. 2020). Long-term clinical follow-up data are still lacking. For example, improvements in insulin signaling and the regulation of daily biological rhythms through fasting may be more pronounced in later years, when both of these functions tend to decline (Luiten et al. 2020; Amiri et al. 2024). Findings from animal studies investigating the timing of fasting onset are inconsistent. In rodents, a delay in starting reduces benefits—those beginning from an early age show extended longevity compared with those starting in adulthood (Goodrick et al. 1982; Mitchell et al. 2019). On the other hand, when applied to young nonhuman primates, fasting is not effective in postponing brain-related illnesses or increasing lifespan (Mattison et al. 2017). To clarify the long-term impact of fasting in humans, especially concerning when to begin, large-scale studies involving various age groups are needed, particularly among older individuals at risk of cognitive decline.

A major issue in applying intermittent fasting to support brain function lies in the absence of clearly established protocols, particularly concerning dietary content, physical activity, and fasting duration. Recommending fasting without addressing these surrounding elements, such as food type, may unintentionally promote poor eating habits. For example, following a 16/8 schedule while consuming ultra-processed or sugar-rich items (which can provoke inflammation) might delay liver energy depletion, thereby postponing fasting-related advantages compared with alternate-day fasting or time-restricted eating paired with nutrient-dense meals. To achieve positive outcomes, intermittent fasting may be more effective when paired with anti-inflammatory, wholesome food selections. This aligns with earlier research involving fasting combined with reduced carbohydrate intake for individuals managing diabetes. Another unknown is whether positive outcomes continue once the fasting plan ends, which is critical for promoting long-term adherence. In the end, integrating multiple healthy lifestyle practices is likely to provide the greatest advantage.

## Future Prospects and Challenges

Future perspectives on aging and health, particularly in the context of IF and cellular autophagy, are promising and multidimensional. With continued research that reveals the complex mechanisms of aging, more emphasis is being put on lifestyle restrictions that can delay age-related deterioration. The function of autophagy, a cellular recycling system that degrades damaged parts, is also better recognized as a key factor in longevity and health.

It is a determinant of pancreatic beta-cell regeneration via autophagy, thus improving insulin secretion as well as glycemic control in diabetic patients. The mechanism involves degradation of Notch1 and upregulation of Ngn3, a key factor in the formation of beta-cells (DiNicolantonio and McCarty 2019). If established in human participants, IF may reverse diabetes (DiNicolantonio and McCarty 2019). Should these findings be confirmed in human subjects, intermittent fasting (IF) may prove to be an effective approach to reversing diabetes (DiNicolantonio and McCarty 2019). Intermittent fasting enhances fatty acid metabolism and mitochondrial function, decreasing oxidative stress. It also leads to the induction of autophagy. Such beneficial effects are potentially useful in preventing neurodegenerative diseases such as Alzheimer’s disease as well as Parkinson’s disease (Quaytman et al. 2024).

Furthermore, future studies will also aim to optimize IF protocols to maximize autophagy to the greatest possible extent, thus leading to improved metabolic health and reducing the impact of age-related disease. In addition, it is also required to understand the synergistic action of genetic as well as environmental determinants on the aging process to devise personalized intervention protocols prepared according to individual specifications. The interplay of biotechnology breakthroughs with nutrition science drives our search for increasing longevity, thus increasing the healthy life span and the free life years from disease. This model has the potential to revolutionize our understanding of aging by shifting attention away from the prolongation of lifespan towards improving quality of life and enhancing well-being in old age (Raza 2024).

However, most of the research on the mechanistic role of IF on autophagy induction and targeting brain aging is based on in-vitro or preclinical models and needs translation to human trials since animal metabolism and responses to fasting vary from that of humans. Apart from this, IF trials on humans that have been done so far include a period of weeks to a few months, which might be insufficient to study the long-term effects on neurodegeneration. Discrepancies in ethnicity, genetic variation, and sex differences also might contribute to varying results and irregularities in the data and therefore must be taken into consideration when performing trials on humans.

## Conclusion

This review highlights the potential advantages of IF, including lower insulin activity, enhanced insulin-like growth factor, better metabolic balance, elevated autophagy, decreased brain inflammation, greater amounts of brain-derived neurotrophic factor, and behavioral improvements. A central theme is the impact of adjustable lifestyle components on aging health. Additional lifestyle factors, such as nutrition, movement, mental activity, and social interaction, have also been linked to increased longevity, increased cognitive resilience, and reduced neurodegeneration (Cohen et al. 2022). Because drugs alone are often ineffective against age-related diseases, researchers suggest combining these modifiable behaviors to promote healthy aging and reduce the severity of neurodegenerative disease risk. Yet, it is still not clear whether intermittent fasting protects the aging brain.

While various human health consequences of IF have been described, further scientific investigation is required to confirm the consequences of food restriction. Among the many unsolved questions is the following: “Fasting decreases inflammation, promotes cleaning of cells, and modulates neurotrophic support pathways to what degree?” Or “Could it be the fasting state itself, and not processes such as inflammation reduction and cleaning of cells, [that] is activating new pathways?” What is the “optimal human fasting method,” and which of the various methods of fasting does it “have the least side effects?” Can such “regimen therapies perhaps cause or be contraindicated in the presence of certain diseases and/or stages of diseases?” What is important is to better understand the biological and biochemical “mechanisms involved” as a means of developing new “therapeutic and preventive approaches.”

In humans, investigations into the impact of intermittent fasting on neurological conditions are relatively rare. Nonetheless, current evidence points to promising outcomes in several disorders. Research findings have established the possible benefits of such methodologies as IF (TRF, PF, ADF, and FMD) as tools for seizure management among patients with epilepsy, improved cognitive function among Alzheimer’s patients, and decreased disability among multiple sclerosis patients, perhaps via modulating microbiota in the gut. (Gudden et al. 2021; Guo et al. 2023). Symptoms related to mood and anxiety may also improve through various proposed biological mechanisms. However, large-scale, randomized, and well-controlled human trials are needed to confirm these early observations (Gudden et al. 2021).

## Data Availability

No datasets were generated or analysed during the current study.

## Abbreviations

IF: Intermittent fasting
SC: Senescent cells
ARD: Age-related diseases
AD: Alzheimer’s disease
PD: Parkinson’s disease
HD: Huntington’s disease
CR: Calorie restriction
SASP: Senescence-associated secretory phenotypes
mTOR: Mammalian target of rapamycin
AMPK: AMP-activated protein kinase
